# Revealing the hidden: A systematic review and meta-analysis of FAPI-based tracers imaging for brain metastatic lesions

**DOI:** 10.1007/s00259-025-07576-6

**Published:** 2025-10-07

**Authors:** Sajjad Sadeghpour, Atena Aghaee, Hojjat Ahmadzadehfar, Alessio Rizzo, Giorgio Treglia, Ramin Sadeghi

**Affiliations:** 1 Nuclear Medicine Research Center, Mashhad University of Medical Sciences, Mashhad, Iran; 2 Department of Nuclear Medicine, Institute of Radiology, Neuroradiology and Nuclear Medicine, University Hospital Knappschaftskrankenhaus, Bochum, Germany; 3 Department of Nuclear Medicine, Candiolo Cancer Institute, Candiolo, TO Italy; 4 Faculty of Biomedical Sciences, Università della Svizzera Italiana, Lugano, Switzerland; 5 Faculty of Biology and Medicine, University of Lausanne, Lausanne, Switzerland; 6 Division of Nuclear Medicine, Imaging Institute of Southern Switzerland, Ente Ospedaliero Cantonale, Bellinzona, Switzerland

**Keywords:** Brain metastasis, FAPI, [^18^F]FDG PET/CT, Cancer, Systematic review

## Abstract

**Background:**

Regarding the assessment of brain metastatic lesions, [^18^F]FDG PET/CT encounters challenges due to heightened physiological uptake in normal brain tissue, resulting in poor tumor-to-background ratios (TBR). Detecting brain metastases accurately has long been a challenge with standard imaging methods. The elevated physiological uptake of [^18^F]FDG in normal brain parenchyma limits its ability to distinguish metastatic lesions owing to poor tumor-to-background contrast. On the other hand, radiolabeled fibroblast activation protein inhibitors (FAPI) have emerged as a newer imaging tracer, showing promise. This study aimed to collect available literature to assess FAPI’s ability to detect brain metastases across various cancer types.

**Methods:**

We reviewed studies published up to April 2025, utilizing different databases, including Google Scholar, PubMed, and Scopus, primarily examining the performance of FAPI based on standardized uptake values (SUVs) and TBRs. Studies focusing on the diagnostic performance of FAPI-based PET for brain metastasis were included. The primary endpoint was the detection rate of FAPI-based tracers.

**Results:**

In 24 studies involving over 115 patients and more than 291 lesions, FAPI-based imaging detected of 87.9% lesions (95% CI: 76.1–94.3%, *p* < 0.001) compared to [^18^F]FDG by the detection rate of 46.3% (95% CI: 30.6–62.8%, *p* = 0.667). The comparison of detection rates of these modalities showed an OR of 10.78 (95% CI: 5.15–22.55, *p* < 0.001). FAPI radiotracers exhibited higher TBR compared to [^18^F]FDG PET/CT (SMD: 1.51, 95% CI: 0.86–2.16, *p* < 0.001).

**Conclusion:**

In conclusion, FAPI**-**based tracers seem to surpass [^18^F]FDG PET in identifying brain metastases. FAPI imaging can potentially serve as a diagnostic tool that offers benefits over traditional methods. Further research is needed to verify its clinical effectiveness.

## Introduction

Brain metastases remain a diagnostic challenge in oncology. Epidemiological data on their true incidence are limited, partly due to underreporting in cancer registries. For example, the Surveillance, Epidemiology, and End Results program records brain metastases only at the time of initial cancer diagnosis, excluding those that develop later [1].

Accurate detection and characterization of brain metastases are crucial for determining the most appropriate treatment strategies, including surgery, radiotherapy, and systemic therapy. The most commonly employed imaging modalities worldwide are magnetic resonance imaging (MRI) and computed tomography (CT).

On the other hand, [^18^F]fluorodeoxyglucose ([^18^F]FDG) positron emission tomography/computed tomography (PET/CT) struggles in brain diagnostics as physiological brain tissue metabolizes glucose, thereby hiding tumors’ uptake. Meanwhile, [^18^F]FDG PET/CT is limited by the high normal brain glucose metabolism, which can make metastatic lesions hard to see because of low tumor-to-background ratios (TBR) [2].

Recently, there has been growing interest in Fibroblast Activation Protein Inhibitor (FAPI)-based PET/CT as a potential means to improve patient management. Early-phase studies employing FAPI-based PET imaging have provided preliminary evidence of its possible utility in detecting brain metastases. This includes both hematogenous spread, as commonly observed in breast and lung cancers, and contiguous extension, such as in head and neck carcinomas, including nasopharyngeal carcinoma [3–5]. Although these promising results are evident, the evidence remains inconsistent, with substantial variability in standardized uptake values (SUVs), lesion sizes, and detection rates across different studies [6, 7].

This systematic review synthesized findings from various studies to evaluate the diagnostic accuracy of FAPI-based imaging for detecting brain metastases, comparing it with [^18^F]FDG PET/CT and MRI. Our objective is to assess the usefulness of FAPI tracers, leveraging TBR, lesion detection rates, and their clinical significance. By synthesizing current evidence, this review seeks to clarify the clinical role of FAPI-based PET/CT and identify areas needing further research.

## Methods

### Study design and data sources

The systematic review was conducted following the PRISMA protocol [8], structured around the following PICO framework: PICO Patient: patients with proven brain metastatic lesions, as detected by another modality (MRI); Intervention: FAPI-based PET/CT imaging; Comparison: other nuclear imaging modalities; Outcome: detection rate.

Comprehensive searches of Medline, Scopus, and Google Scholar were conducted using the keywords: terms (“FAPI” OR “Fibroblast Activation Protein Inhibitor”) AND (“PET” OR “PET/CT” OR “Positron Emission Tomography”) AND (“brain metastasis” OR “brain metastases” OR “cerebral metastasis” OR “CNS metastasis”). No restrictions on language or publication date were applied. Only studies focused on the FAPI-imaging compared to other nuclear modalities were included. To ensure thorough coverage, reference lists and citing articles of the retrieved studies were examined for additional relevant studies. The last search was completed in April 2025.

Eligibility criteria required that human studies report sufficient data to extract the detection rate, SUV, and TBR of FAPI-based imaging. The reviews, editorials, case reports, and conferences were excluded. In cases of duplicate studies, only the most recent or comprehensive publication was included.

### Data extraction

We used a standardized form to extract things like patient numbers, cancer type, imaging results, and how (or if) the imaging changed treatment. From there, we pooled the data and looked for trends in performance between FAPI and [^18^F]FDG PET/CT.

The Risk of Bias (RoB) for the included studies was assessed using the QUADAS-2 checklist for diagnostic accuracy studies [9]. This tool evaluates areas such as patient selection, the index test, the reference standard, and flow and timing. Each domain was examined for potential bias, with the first three also considering applicability concerns. The preferred reference standard was either biopsy-confirmed results or MRI, regarded as the gold standard for diagnosis.

### Synthesis of results

Meta-analyses of sensitivity and detection rate were conducted with a random-effects model (Der-Simonian and Laird method). This method considers variability between studies, making it ideal for combining data from studies with diverse features. Heterogeneity was evaluated using the Cochrane Q test, with significance defined as *p* < 0.05, and measured with the I^2^ index, which indicates the proportion of variability due to actual heterogeneity rather than sampling error.

Publication bias was assessed through funnel plots, Egger’s regression intercept, and the Duval–Tweedie trim and fill method. Funnel plots show the standard errors of included studies on one axis and their effect sizes on the other. Asymmetry in these plots indicates potential publication bias, quantified by Egger’s regression intercept (*p* < 0.05 reveals significant bias). The Duval–Tweedie trim and fill method corrects for this bias by removing smaller studies iteratively to restore symmetry, providing an adjusted pooled effect size to estimate the influence of publication bias. Meta-analytical calculations were carried out using Comprehensive Meta-Analysis (version 2). Key diagnostic indices included detection rate, SUV, and TBR.

## Results

### General data of included studies and risk of bias assessment

As both the FAPI, [^18^F]F-labelled and [^68^Ga]Ga-labelled radiotracers were intended and evaluated during this study, for the sake of simplicity, the more general terms “FAPI-based tracers” and “FAPI-based imaging” were used.

**Fig. 1:**
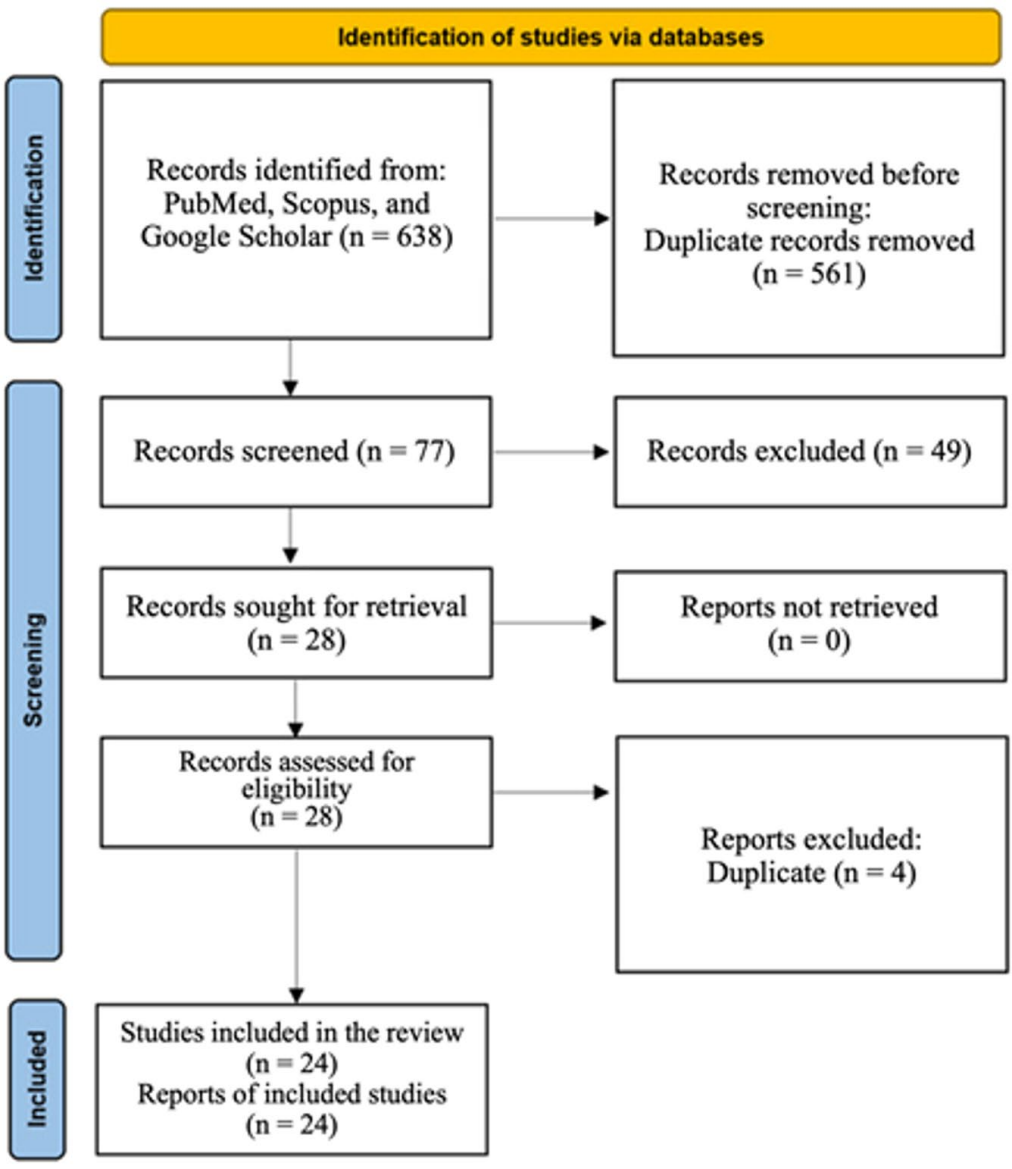
PRISMA flow diagram

Of the 644 primary papers found, 28 were selected for inclusion. Similar studies, conducted in similar centers with comparable recruitment times, were evaluated for possible overlap. Of the 28 studies, four were excluded due to overlapping data, and patients were excluded from the study [10–13]. Of the four papers authored by Ballal et al. [4, 14–16], all were investigated regarding overlap; in terms of different cancer types and recruitment time, possible overlap was ruled out. Three additional studies, authored by Qiu et al. [17], Wu et al. [7], and Liu et al. [18], were investigated, and the possible overlap was ruled out (Fig. 1).

This systematic review and meta-analysis evaluated the diagnostic efficacy of FAPI compared to [^18^F]FDG PET in detecting brain metastatic lesions. This study synthesizes data from 24 studies assessing the effectiveness of FAPI-based PET/CT in detecting brain metastatic lesions. Studies were conducted in China (*n* = 16), followed by India (*n* = 4), Turkey (*n* = 2), Thailand (*n* = 1), and Iran (*n* = 1). The research designs included prospective (*n* = 16) and retrospective (*n* = 8) studies, focusing on primary tumors such as lung, breast, nasopharyngeal, and thyroid cancers. Patients with brain metastases ranged from 1 to 17, with lesion counts from 1 to 76 per study. Overall, over 115 patients and more than 291 brain lesions were evaluated. Primary outcomes included detection rate, SUV, and TBR (Table 1).

**Table 1:** Characteristics of the included articles

First author	Compared radiotracers	Sample size (patients)	Primary tumor type	Average SUV_max_ (FAPI vs. comparison)	Average TBR	Other Findings	Limitations
Qiu [17]	[68Ga]Ga-FAPI-04, [18 F]FDG	2	n.a.	5.55 vs. 8.70	Not available	FAPI PET changed staging in one patient with brain metastases missed by FDG. Superior TBR for brain metastases.	No specific limitations mentioned.
Ballal [4]	[18 F]FDG, [68Ga]Ga-DOTA.SA.FAPi	12	7 breast cancer, 1 SCC, 1 multiple myeloma, and 3 lung cancer	SULpeak: 10.1(5.7–14.9) vs. 7.7(5.1–9.8)/SULave: 8(4.6–13.9) vs. 5(4.2–6.8)	FAPI: T/parenchy: peak 832 (34.2–249.3.2.3) vs. 1.5(1–2.3.3)/average: 59.3(33.5–130.8.5.8)vs 1.2(1–2.3.3)	FAPI identified additional brain lesions in 2 patients potentially altering treatment planning. 2% discordance between modalities. FAPI may detect occult brain metastases missed by FDG.	No specific limitations mentioned.
Zhao [5]	[18 F]FDG, [68Ga]Ga-FAPI	4	Nasopharyngeal carcinoma	n.a.	n.a.	FAPI detected additional lesions including skull base/intracranial invasion leading to TNM stage change.	Small sample size. no histopathological validation.
Zhou [19]	[68Ga]Ga-DOTA-FAPI-04, [18 F]FDG.	4	Lung cancer (NSCLC)	3.1 ± 1.7	n.a.	No specific findings on brain metastases limited data.	Limited number of brain metastases. No detailed SUV or TBR comparison.
Ballal [16]	[68Ga]Ga-DOTA.SA.FAPi, [18 F]FDG	11	Radio-iodine-refractory Follicular thyroid carcinoma	SULpeak: 13.9 (6.89 to 28.71) vs. 6.7 (2.24 to 9.23)//SULav:6.89 (2.1 to 9.23) vs. 3.23 (1.1 to 6.72)	n.a.	Improved detection of brain metastases led to altered therapy in 7 patients (18%). FDG missed 2 patients and 14 lesions.	No histopathology for brain lesions heterogeneous tumor subtypes.
Zheng [20]	[68Ga]Ga-DOTATATE, [68Ga]Ga-FAPI	2	Nasopharyngeal carcinoma	7.6 ± 2.8 vs. 5.2 ± 1.9	n.a.	FAPI and DOTATATE equally detected brain metastases.	Small sample size for brain metastases. No histopathological confirmation.
Tian [21]	[18 F]F-FAPI-04, [18 F]FDG	5	n.a.	2.5 ± 2 vs. 7.6 ± 1.8	25.3 ± 20.1 vs. 0.7 ± 0.2	FDG missed 7 brain metastases; FAPI superior due to low brain background. Led to treatment adjustments in 17% of patients. Low FDG uptake in small lesions.	Lack of histopathological confirmation for some lesions.
Kömek [22]	[68Ga]Ga-FAPI-04, [18 F]FDG	1	Breast cancer	n.a.	n.a.	FDG missed all brain metastases; FAPI detected all.	No specific limitations mentioned (MRI confirmation used).
Mu [23]	[18 F]F-FAPI-42, [18 F]FDG	10 lesions	Lung cancer	3.3 (FAPI)	n.a.	FAPI less sensitive for brain metastases compared to other sites.	No specific limitations mentioned.
Zhao [24]	[68Ga]Ga-FAPI-RGD, [68Ga]Ga-FAPI-46, [18 F]FDG	1	n.a.	RGD:5.8 vs. FAPI-46:3 vs. [18 F]FDG:6.6	RGD:143.8 vs. FAPI-46:83.7	Improved delineation of brain metastases for radiotherapy planning.	Small sample size no direct comparison with MRI.
Radmehr [25]	[68Ga]Ga-FAPI-46, [18 F]FDG	1	Breast cancer	3.09	1.8 (FAPI)	Potential for improved detection of small metastases including brain.	Very small sample size. No MRI correlation.
Wu [7]	[68Ga]Ga-DOTA-FAPI-04, [18 F]FDG	1	DTC	0.89 vs. 2.08	higher for FAPI	Improved detection of small brain metastases.	Limited brain metastasis cases.
Liu [18]	[68Ga]Ga-FAPI, [18 F]FDG	12	9 Lung cancer, 1 ovarian cancer, 1 DTC, 1 melanoma	2.1 (0.01–17.01) vs. 7.9 (2–17.9.9)	27.8 (1–230) vs. 1 (1–2.1.1)	Better delineation of lesions for therapy planning.	Small sample size.
Ballal [15]	[68Ga]Ga-DOTA-SA-FAPI, [18 F]FDG	15	Breast cancer	5.3(4.3–6.8) vs. 3.2(1.7–3.9)	22.3(11.6–35.6) vs. 6.8(1.9–9.8)	Improved visualization of brain metastases, aiding in treatment planning.	Lack of histopathology for all lesions.
Hua [26]	[68Ga]Ga-FAPI-04, FET	3	n.a.	5 vs. 2.6	160 vs. 7	Potential for differentiating tumor types via MTVFAPI: MTVFET ratio.	Small sample size lack of standardized delineation guidelines.
Can [27]	FDG, Ga-FAPI-04, MRI	1	NSCLC	n.a.	n.a.	-	Retrospective design, small sample, no surgical correlation for lesion.
Miao [28]	Ga-FAPI-04, [18 F]FDG	1	Esophageal SCC	n.a.	n.a.	FAPI PET/MRI outperforms 18 F-FDG in detecting atypical metastases.	Small sample; focus on GI cancers limits brain metastasis data.
Wang [29]	[68Ga]Ga-FAPI-RGD, [18 F]FDG, [68Ga]Ga-RGD, [68Ga]Ga-FAPI	1	Lung cancer	5.6 vs. 7.7	n.a.	Improved detection of brain metastases (skull metastasis SUVmax 15.2 vs. no uptake on FDG).	Small sample size.
Wang [6]	[68Ga]Ga-FAPI vs. [18 F]FDG	23 lesions	Lung cancer	9 vs. 7.4	314.4 vs. 1	FAPI outperformed FDG in detecting brain metastases but MRI superior. Variable FAPI uptake. High inter-reader agreement for FAPI (k = 0.9). Low FAPI uptake (SUVmax ≤ 2.5) in 48% (11/23) of brain lesions.	Lack of histopathologic confirmation small sample size low FAPI uptake in some lesions.
Wei [30]	[18 F]F-FAPI, [18 F]FDG	8	Lung cancer	n.a.	n.a.	Fluorine 18-FAPI PET/CT was found to be superior to FDG for detecting metastatic lung cancer, especially in brain. FAPI PET/CT may help improve the accuracy of lung cancer staging.	Lack of histopathologic confirmation, small sample size.
Li [31]	[18]FAPI, [18 F]FDG	17	Lung adenocarcinoma	1.56 ± 2.19 vs. 7.34 ± 3.54	9.53 ± 12.07 vs. 1.01 ± 0.49	TNM stage upgraded in 4 patients due to FAPI-detected metastases.	Small sample size. Few early-stage lung cancer patients included. Pathological confirmation not available for all lesions.
Yang [32]	[¹⁸F]F-FAPI-04 and [¹⁸F]F-FDG	7 lesions	GI (gastric, pancreatic, liver, colorectal) cancers	3.8 vs. 3.2	9.6 vs. 2.5	Revised diagnosis in 31.7% of patients; management changes in 21.7%.	Heterogeneous population. No pathological confirmation for all lesions.
Siripongsatian [33]	[68 Ga]Ga-FAPI-46, [18 F] FDG	1	cholangiocarcinoma	25.3 vs. 8.86	194.6 vs. 1.16	FAPI identified a brain metastasis missed by FDG altering radiotherapy planning.	Single case of brain metastasis no histopathology correlation.
Ballal [14]	[68Ga]Ga-DOTA.SA.FAPi, [68Ga]Ga-DOTANOC	3	MTC	SUL: 6 (3.4–6.9) vs. 4.2(2.1–6.2)	(SUV peak-based): 12 (1.9–14.2) vs. 8 (5.2–10.9)	-	Small sample size. No histopathology validation (validated with diagnostic CT).

Most studies demonstrated low RoB in the domains of patient selection and index test. The reference standard domain generally showed a low risk, although some studies raised moderate to high concerns. Flow and timing were a common area of concern, with several studies exhibiting high RoB in this domain. Applicability concerns were generally low across domains; however, notable exceptions were observed, which had high concerns for the index test. Overall, while many studies maintained a low RoB across domains, there remains a subset with methodological limitations warranting cautious interpretation of their findings (Table 2).

**Table 2:** Risk-of-bias of the included studies using the QUADAS-2 checklist

First author	Patient selection		Index test		Reference standard		Flow and timing
	Risk of bias	Applicability concern	Risk of Bias	Applicability concern	Risk of bias	Applicability concern	Risk of bias
Qiu [17]	low	High	low	Low	high	High	low
Ballal [4]	low	Low	low	Unclear	Unclear	Low	low
Zhou [19]	low	low	low	low	low	low	low
Ballal [16]	Low	Low	low	low	Unclear	Low	low
Zhao [5]	Low	low	low	low	Low	Low	low
Zheng [20]	low	high	low	high	low	High	low
Tian [21]	low	Low	low	low	unclear	low	Low
Kömek [22]	low	Low	low	high	low	high	low
Mu [23]	low	Low	low	high	low	high	low
Can [27]	Low	High	low	high	Low	Low	low
Zhao [24]	low	High	low	high	low	high	low
Radmehr [25]	low	High	low	high	Low	Low	Low
Wu [7]	Low	High	Low	Low	Low	Low	Low
Liu [18]	low	Low	Low	high	Low	Low	Low
Ballal [15]	low	Low	Low	Low	High	High	Low
Hua [26]	Low	High	Low	Low	Low	Low	Low
Miao [28]	Low	High	Low	Low	Low	Low	Low
Wang [20]	Low	High	Low	Low	Unclear	Low	Low
Wang [6]	High	Low	Low	Low	Unclear	Low	Low
Wei [30]	Low	Low	Low	High	Low	High	Low
Li [31]	low	Low	Low	High	Low	High	Low
Yang [32]	low	Low	Low	Low	Unclear	Low	Low
Siripongsatian [33]	High	High	Unclear	Low	Low	Low	Low
Ballal [14]	Low	Low	Low	Low	Low	Low	Low

### Detection rate comparison

Eleven studies were included in the meta-analysis to calculate the detection rates of FAPI and [^18^F]FDG for brain metastatic lesions. The pooled detection rate for FAPI-based imaging was 87.9% (95% CI: 76.1–94.3%, *p* < 0.001). Heterogeneity analysis indicated a Cochrane Q value of 27.64 (*p* = 0.002) and an I^2^ index of 63.82%. On the other hand, the [^18^F]FDG pooled detection rate was calculated as 46.3% (95% CI: 30.6–62.8%, *p* = 0.667), with a Cochrane Q value of 42.16 and an I^2^ index of 76.28%.

**Fig. 2:**
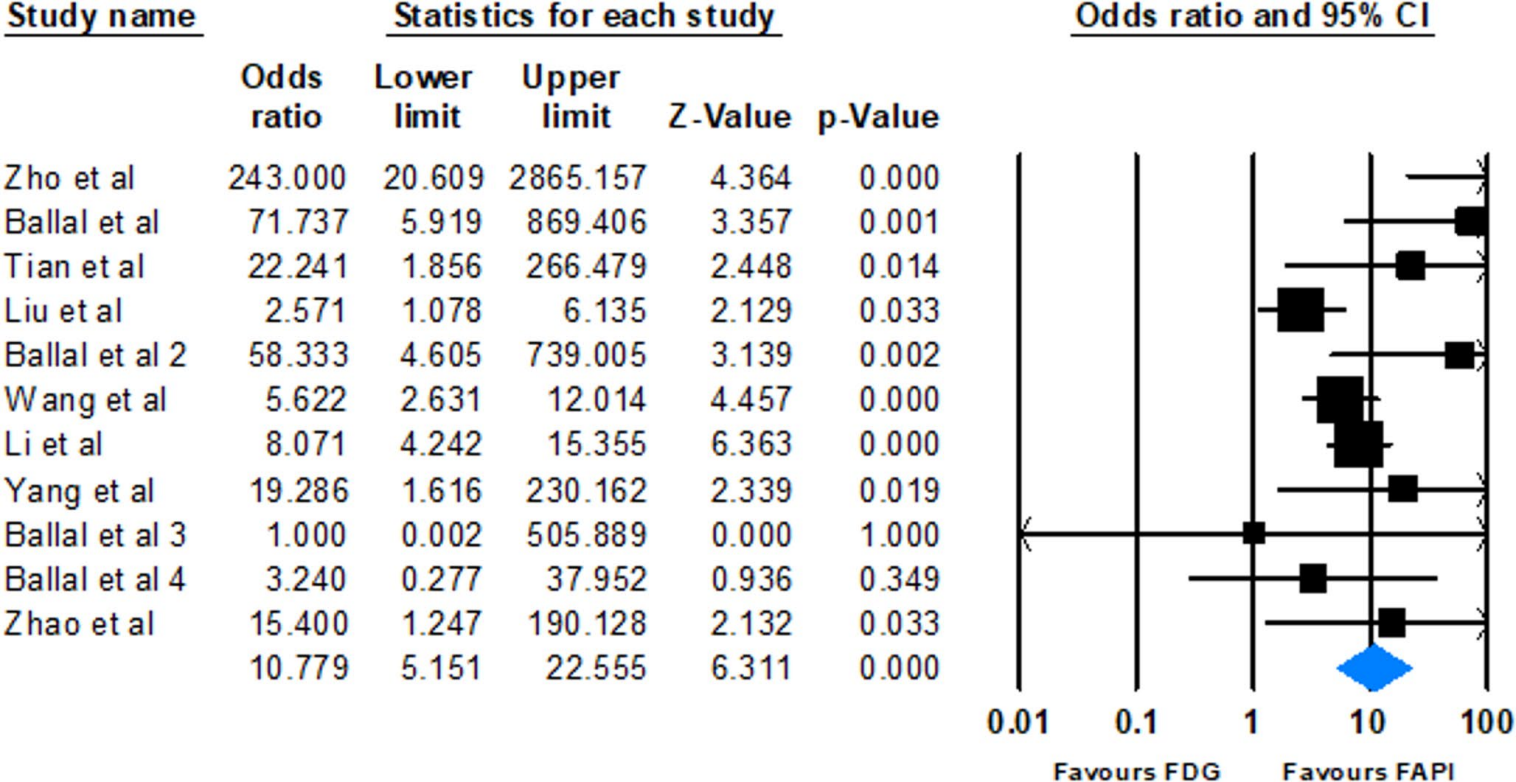
Forest plot illustrating the pooled odds ratios of lesions detection by FAPI as compared to FDG across included studies

The odds ratios from individual studies ranged from 1.000 to 243.000, mostly indicating a higher detection rate of FAPI compared to [^18^F]FDG. The combined analysis using a random-effects model revealed that FAPI had a notably higher detection rate, with a pooled odds ratio of 10.779 (95% CI: 5.151–22.555) and a p-value of less than 0.001 (Fig. 2).

**Fig. 3:**
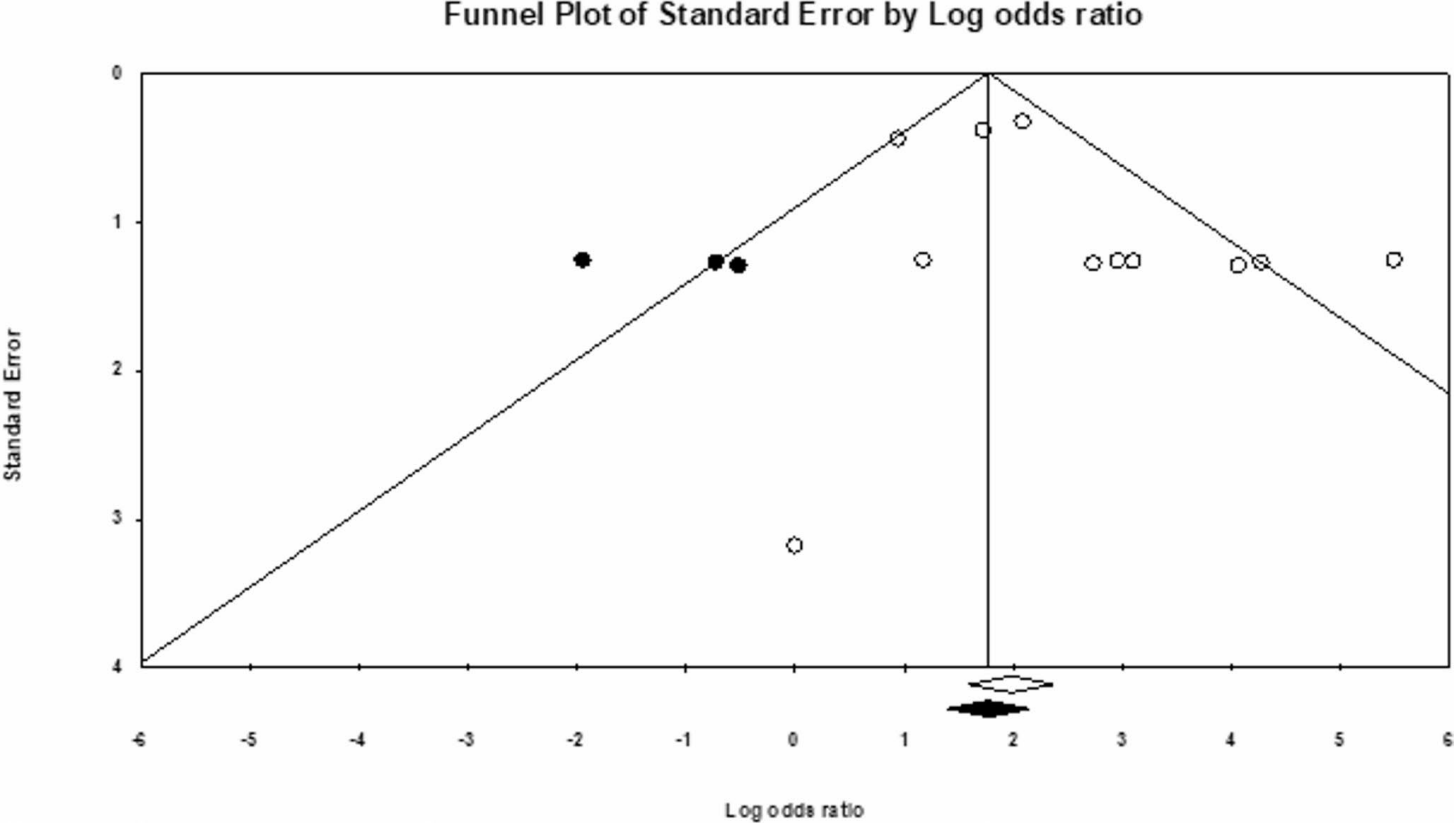
Funnel plot of detection rate OR pooling. White circles represent the included studies, and a white diamond indicates the pooled detection rate of these studies. Black circles represent the studies trimmed to correct for asymmetry. In contrast, the black diamond represents the adjusted pooled effect size accounting for potential publication bias, as calculated using the Duval–Tweedie trim and fill method

Publication bias was assessed through funnel plots and statistical methods. Analysis of publication bias using Egger’s test and adjustment with trim-and‐fill did not significantly change the overall Odds Ratio (OR), indicating small-study effects are unlikely to influence the outcome. Egger’s regression intercept was calculated at 1.25 (*p* = 0.11). After applying Duval–Tweedie’s trim and fill method and removing three studies, the funnel plot achieved symmetry, resulting in an adjusted pooled OR of 6.38 (95% CI: 2.83–14.38), reflecting a 4.4 reduction from the original pooled OR (Fig. 3).

### SUV comparison

**Fig. 4:**
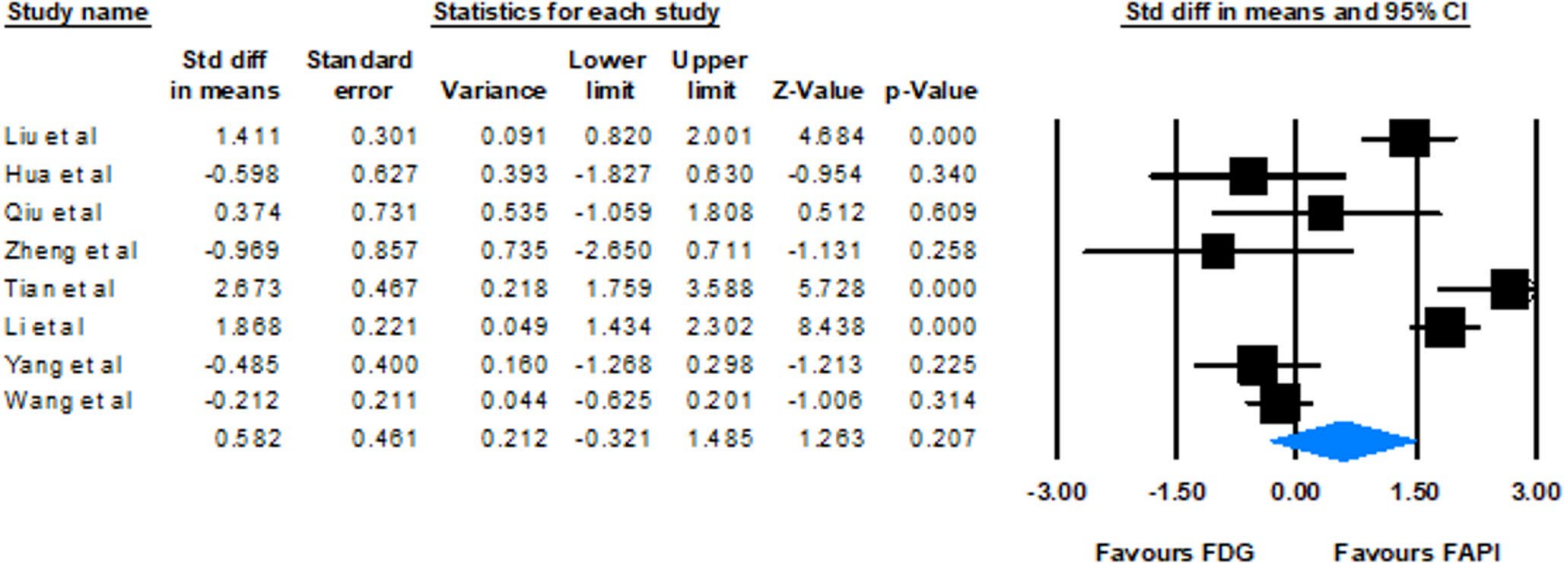
Forest plot illustrating the pooled standardized difference in mean SUV of FDG compared to FAPI across included studies

Eight studies reported SUVs for FAPI and [^18^F]FDG, assessing the contrast between lesion uptake and background tissue. The standardized mean differences (SMD) varied considerably, from − 0.969 to 2.673, indicating heterogeneity. Studies by Liu et al. [18], Tian et al. [34], and Li et al. [31] reported notably higher SUV for FAPI, while others showed no significant difference or favored FDG. The random-effects model pooled SMD was 0.582 (95% CI: −0.321 to 1.485, *p* = 0.207). Heterogeneity analysis revealed a Cochrane Q value of 86.44 (*p* < 0.001) and an I^2^ index of 91.90%. These can indicate a tendency toward higher lesion uptake with FAPI, but the confidence intervals cross zero (Fig. 4).

**Fig. 5:**
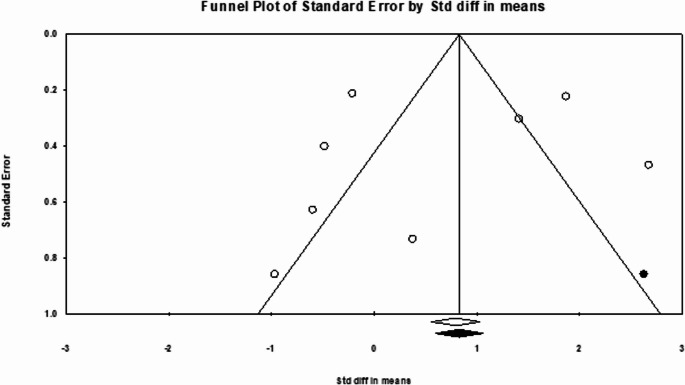
Funnel plot of SUV pooling. White circles represent the included studies, and a white diamond indicates the pooled detection rate of these studies. Black circles represent the studies trimmed to correct for asymmetry. In contrast, the black diamond represents the adjusted pooled effect size accounting for potential publication bias, as calculated using the Duval–Tweedie trim and fill method

For SUV SMD, the funnel plot depicted in Fig. 5 also showed asymmetry, with Egger’s regression intercept at 1.23 (*p* = 0.69). Following the removal of one study through the trim and fill method, a symmetrical funnel plot was obtained. The adjusted pooled SUV SMD was 0.76 (95% CI: −0.10 − 1.63), reflecting a 0.18 increase from the observed pooled SUV SMD.

### TBR comparison

Eight studies provided data on TBR values for both FAPI and [^18^F]FDG, assessing the intensity of tracer uptake in brain metastatic lesions. The SMD ranged from 0.471 to 5.234 across the studies, all indicating higher TBR values for FAPI. The meta-analysis, using a random-effects model, found a pooled SMD of 1.513 (95% CI: 0.863–2.163, *p* < 0.0001); FAPI shows significantly higher TBR values. Heterogeneity analysis revealed a Cochrane Q value of 62.39 (*p* < 0.001) and an I^2^ index of 88.78%.

**Fig. 6:**
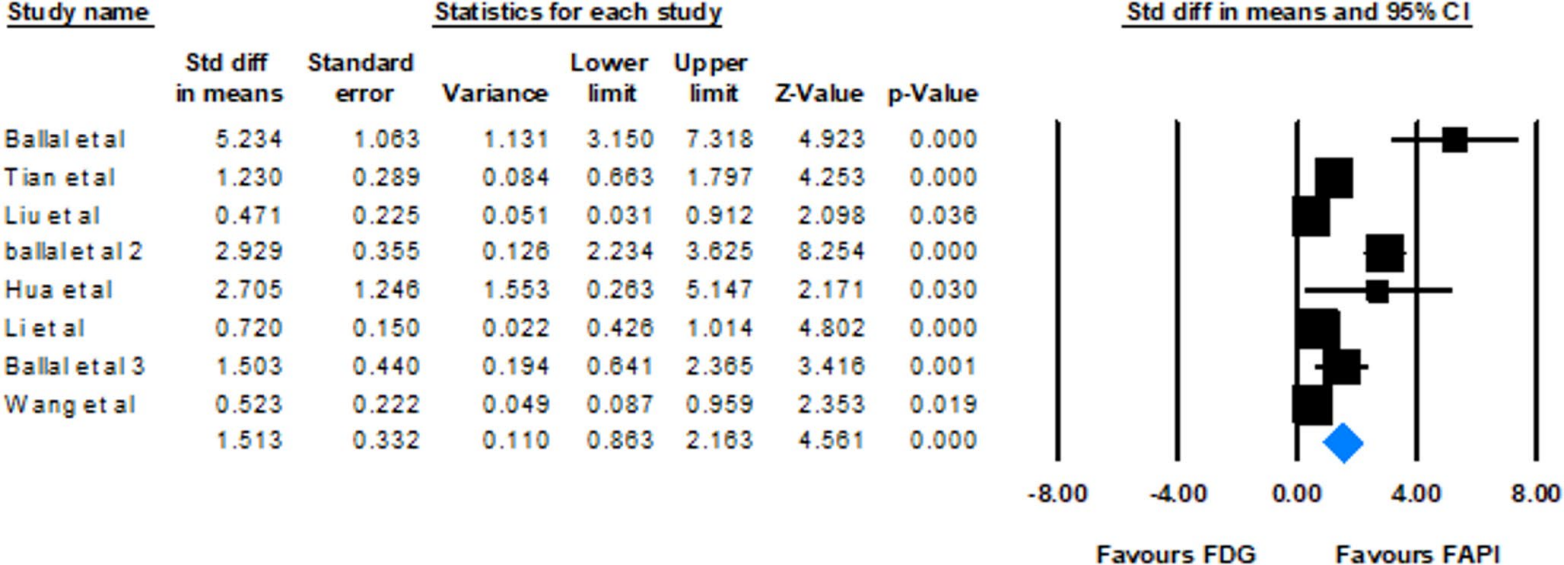
Forest plot illustrating the pooled standard mean TBR differences of FDP and FAPI across studies

**Fig. 7:**
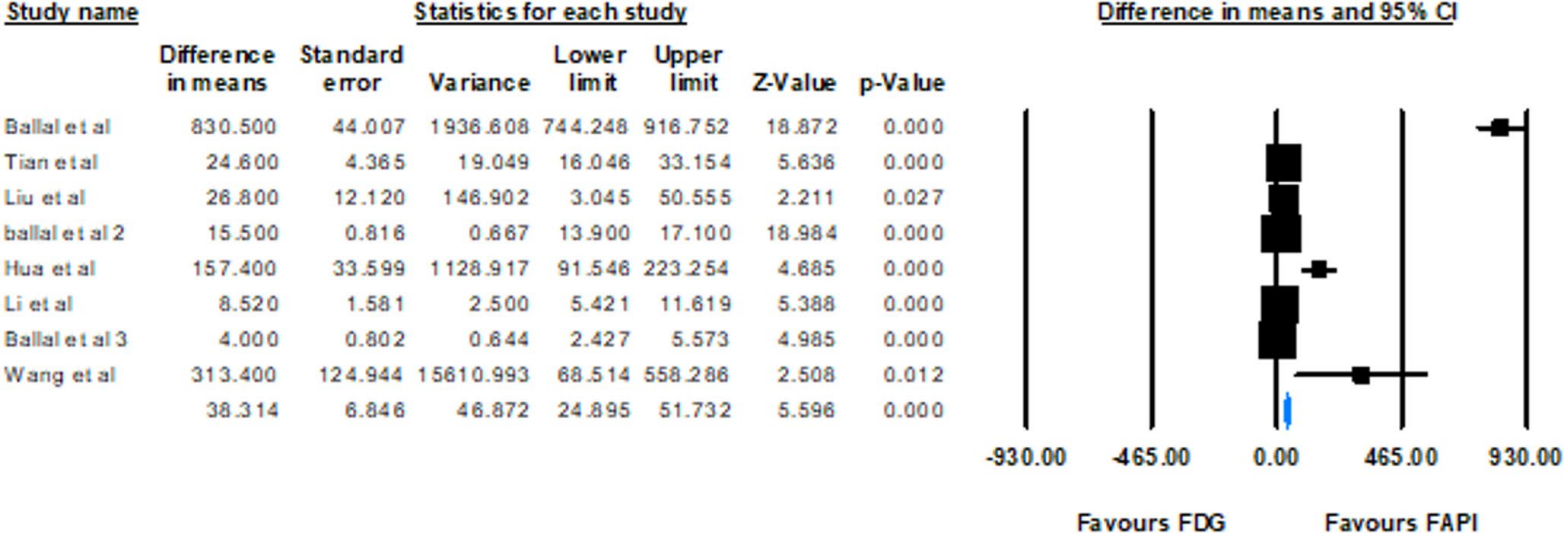
Forest plot illustrating the pooled TBR mean differences of FDG and FAPI across studies

The analysis of the difference in means for TBR revealed values ranging from 4.000 to 830.500 across different studies. The combined mean difference was 38.314 (95% CI: 24.895–51.732, *p* < 0.001), supporting that FAPI has a higher TBR than FDG. Of particular note, the study by Ballal et al. showed an unusually high difference in means of 830.500, which could be an outlier or specific factors affecting this result (Figs. 6 and 7).

**Fig. 8:**
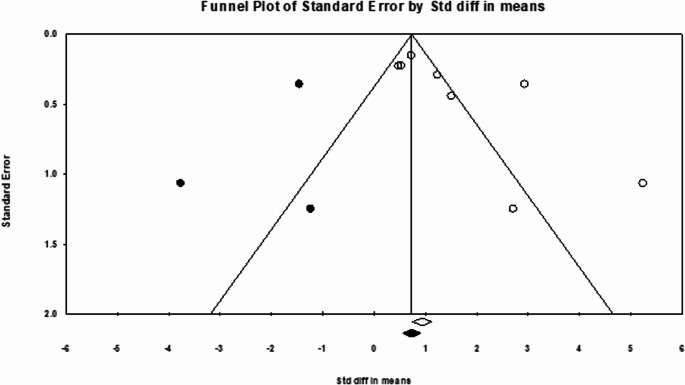
Funnel plot of SUV pooling. White circles represent the included studies, and a white diamond indicates the pooled detection rate of these studies. Black circles represent the studies trimmed to correct for asymmetry. In contrast, the black diamond represents the adjusted pooled effect size accounting for potential publication bias, as calculated using the Duval–Tweedie trim and fill method

Regarding TBR, the funnel plot depicted in Fig. 8 showed asymmetry, with Egger’s regression intercept at 4.08 (*p* = 0.04). Following the removal of three studies through the trim and fill method, a symmetrical funnel plot was obtained. The adjusted pooled TBR SMD was 0.81 (95% CI: 0.06–1.55), reflecting a 0.7 reduction from the observed pooled TBR SMD.

### Other findings

Brain metastatic lesions detected by FAPI-based PET/CT ranged from 0.53 cm to 3.05 cm. Liang Zhao et al. [24]. identified lesions as small as 0.4 cm. In comparison, Halil Kömek [22] reported lesions up to 8 cm. Ya Liu [18] noted lesions ranging from 0.53 to 1.64 cm. Lijuan Wang [6] reported an average lesion size of 1.3 cm for 23 lesions, demonstrating the FAPI-based tracer’s ability to detect both small and large metastases.

The efficacy of FAPI-based PET and [^18^F]FDG PET/CT in detecting brain metastatic lesions varied depending on the type of primary tumor. In lung cancer, FAPI-based PET/CT demonstrated a significantly higher detection rate for brain metastases compared to [^18^F]FDG PET/CT, as evidenced by Xiang et al. in their study [10], which showed that FAPI-based tracers detected 23 brain metastases compared to 10 detected by [^18^F]FDG PET/CT. Similarly, for breast cancer, even though FAPI-based PET/CT was superior in identifying cerebral metastases, their overall diagnostic performance was closer [15].

Finally, beyond better visualizing the lesions and improved detection rate, FAPI-based imaging could alter the TNM staging and treatment planning. FAPI-based PET/CT influenced clinical management in several studies; in a survey, the TNM stage was upgraded in 4/17 patients due to FAPI-based imaging detecting metastases [31]. Another study [33] reported altered radiotherapy planning due to the detection of a brain metastasis. Another study reported [32] revised diagnoses in 31.7% of patients and management changes in 21.7%. Sanjana Ballal [15] noted that improved visualization aided treatment planning. These findings suggest FAPI-based imaging can provide actionable information, potentially impacting staging, treatment decisions, and patient outcomes (Table 2).

## Discussion

### Fibrotic activation and tumour microenvironment

Fibroblast Activation Protein (FAP) is overexpressed in cancer-associated fibroblasts (CAFs) within the tumor stroma. CAFs support tumor-promoting functions, including angiogenesis, cell proliferation, chemoresistance, extracellular matrix remodeling, and immunosuppression [35]. The tumor microenvironment (TME) impacts how therapies work, with CAFs playing a key role in certain tumors. FAP-positive CAFs modulate the immune landscape by secreting immunosuppressive cytokines and chemokines, leading to the recruitment of cells that suppress immune responses [36].

False positives can occur due to FAPI uptake in conditions like fibrosis or inflammation after surgery or radiation therapy [36, 37], complicating image interpretation, especially when evaluating residual cancer shortly after chemotherapy [38]. In the context of brain metastatic lesions, progressive multifocal leukoencephalopathy and tuberculosis meningitis are prototypical examples [39, 40].

### Superior detection rate of FAPI-based imaging

The results of the meta-analysis indicated a detection rate of 87.9% (95% CI: 76.1–94.3%) for FAPI-based imaging. However, the detection rate for [^18^F]FDG was 46.3% (95% CI: 30.6–62.8%). FAPI-based imaging significantly outperforms [^18^F]FDG in detecting brain metastatic lesions. The pooled OR of 10.779 (95% CI: 5.151–22.555, *p* < 0.001) from a random-effects model demonstrates a significantly higher detection rate for FAPI, indicating that FAPI-based imaging was 10.77 times more likely to diagnose brain lesions compared to [^18^F]FDG. Overall, the observed higher detection rate of FAPI-based imaging can be attributed to the better lesion visualization and contrast of FAPI.

Although heterogeneity was observed among studies (I² = 54.8%), suggesting some variability in effect size, the consistent trend across individual studies, with most ORs favoring FAPI, shows the reliability of this. The consistency of these findings is supported by the publication bias, which, via Egger’s test and trim-and-fill adjustment, did not materially change the pooled OR, indicating that small-study effects do not drive the observed results.

### Varied SUV comparisons and lesion size detection

The comparison of SUV values between FAPI and [^18^F]FDG presented more varied results, with SMD ranging from − 0.969 to 2.673. The random-effects model SMD was 0.582 (95% CI: −0.321 to 1.485, *p* = 0.207), indicating no statistically significant difference in SUV between FAPI and [^18^F]FDG. The lack of statistical significance in the random-effects model, despite a tendency toward higher FAPI SUVs, may be due to overlapping confidence intervals in some studies. Of note, some studies have reported significantly higher SUVs for FAPI. However, others showed no significant difference or even favored [^18^F]FDG, reflecting the complex interaction between tracer uptake and background activity.

Based on the considerable heterogeneity in evaluating SUV (*I*² = 91.90%), its value was mainly driven by primary tumor type. Variations in tracers added further inconsistency: FAPI-RGD had a higher uptake in a head-to-head comparison with FAPI-46, owing to differences in target affinity, biodistribution, blood-brain barrier (BBB) transmission, etc [24]. Tumor stage also played a role, as advanced tumors showed higher uptake due to more fibrosis; early-stage metastases risked underdetection with lower SUV [6, 31]. Additionally, diverse imaging protocols such as post-injection timing, activities, scanner types, and reconstruction methods can further increase heterogeneity.

Regarding the primary tumor type and tumor differentiation, as shown, breast cancers [15], Medullary Thyroid Carcinoma (MTC) [14], GI (gastric, pancreatic, liver, and colorectal) cancers [32], nasopharyngeal cancers [20], and Follicular Thyroid Carcinoma (FTC) [16]can have higher FAPI-based tracer uptake. However, in cancers such as cholangiocarcinoma [33], the [^18^F]FDG SUV is higher. Metastases from stromal-rich cancers often had higher SUVmax values due to increased FAP expression in the TME. Additionally, some cancers, such as lung cancer, display a dual behavior; in some studies, they have higher FAPI-based tracer uptake, and in others, they show higher [^18^F]FDG uptake [6, 29, 41]; this can be explained by the lung subtype cancers (such as small cell, large cell, adenocarcinoma) and their cellular uptake of the FAPI-based tracers. Another important note is that the FAPI-based tracers’ uptake is the lowest among different sites of metastases; this can be attributed to the BBB [31].

### TBR as a key indicator

The analysis of TBR values provides convincing evidence for the diagnostic advantage of FAPI-based imaging over [^18^F]FDG. Data from eight studies were used to compare the TBR values; all reported higher TBR values for FAPI, with SMD ranging from 0.471 to 5.234. The pooled SMD of 1.513 (95% CI: 0.863–2.163, *p* < 0.0001) from a random-effects model confirms that FAPI exhibits significantly higher SUV values relative to background compared to [^18^F]FDG. These estimates greatly exceed zero, indicating that FAPI uptake in lesions is substantially elevated compared to the surrounding normal brain tissue, which typically has low FAPI uptake, unlike the high physiological uptake of [^18^F]FDG in the brain parenchyma. This high contrast significantly improves the visualization and delineation of brain lesions, which is a major limitation of [^18^F]FDG PET/CT in brain imaging [42]. The strong significance of the pooled results, even under random-effects pooling, reinforces the clinical utility of FAPI’s elevated TBR. The superior TBR of FAPI allows for clearer visualization of lesions, even small ones, potentially leading to earlier detection and more accurate characterization of brain metastases. For instance, one study highlighted that while lesions showed higher [^18^F]FDG uptake, FAPI PET was superior in delineating cerebral lesions due to the low background FAPI activity in the brain parenchyma [31].

Heterogeneity (I² = 88.8%) indicates variability among the included studies. As already stated, primary tumor types influenced this variation, with metastases from cancers like breast cancer, advanced lung cancers, and cholangiocarcinoma often showing high TBRmax [4, 6, 33]. In contrast, MTC, GI tumors (gastric, pancreatic, liver, and colorectal), and lung adenocarcinoma had lower uptake [14, 31, 32]. Different pharmaceutical forms enhanced the inconsistency index: FAPI-RGD showed higher contrast compared with FAPI-46, possibly due to biodistribution and clearance. Futhermore, tumor stage also mattered; as advanced lesions showed better contrast due to fibrosis, while early metastases had lower TBR; considering lung cancer with the similar subtypes, advanced disease showed significantly higher TBR compared to the primary staging [6, 31].

### Impact on TNM staging and clinical management

The improved lesion detection and higher detection rates offered by FAPI-based imaging have significant implications for cancer staging and treatment planning, especially when comparing FAPI versus [^18^F]FDG in brain metastatic lesions. A study found that FAPI-based PET/CT altered the TNM stage in 11.4% of patients, with the majority of these changes resulting in upstaging, indicating a more accurate assessment of disease extent [31]. Changes in clinical management occurred in several patients [31–33]. The possible considerable changes can include irradiation of new brain lesions, a shift from local to systemic therapy, or adjustments in treatment intent (from curative to palliative). The enhanced lesion delineation in radiation therapy planning, which can potentially lead to reduced off-target exposure and improved dosimetry, represents an important clinical benefit. The ability of FAPI-based imaging to provide actionable data that directly affects staging and treatment decisions underscores its ability to enhance patient outcomes.

The ability of FAPI-based tracers to detect both small and large metastases, ranging from 0.53 cm to 5.8 cm in diameter, underscores their versatility and diagnostic potential. This is particularly important when [^18^F]FDG has limitations in detecting smaller lesions, particularly those less than 1 cm in diameter, due to high parenchymal physiological uptake [34].

### Comparison with other modalities

Although FAPI-based imaging has demonstrated superior diagnostic accuracy relative to [^18^F]FDG PET/CT, the current gold standard for brain imaging remains MRI. In a study [6], MRI identified a greater number of lesions than FAPI-based imaging, suggesting that the latter may have limited sensitivity for detecting small-volume or early-stage brain metastases.

MRI limitations include low specificity in differentiating neoplastic tissue from treatment-related changes, such as pseudoprogression or radionecrosis. It also faces challenges in accurately defining lesion boundaries and is prone to artifacts from metallic implants or skull base, which can impair precise comparisons [18, 43, 44]. Additionally, contrast-enhanced MRI provides only an approximate measure of BBB permeability, without offering a quantitative assessment, which may limit its usefulness as a reference in heterogeneous tumors [18].

Nevertheless, FAPI-based PET offers unique advantages, notably its ability to provide functional information alongside whole-body assessment in a single examination, which is particularly valuable for comprehensive oncologic staging.

### Clinical implications of biological mechanisms and the role of the BBB

In many cancer types, tumor-associated fibrosis and elevated FAP expression are associated with advanced disease, poorer prognosis, and worse clinical outcomes [45–47].

FAPI uptake in brain metastatic lesions indicates stromal activity and might provide valuable insights into therapy and prognosis [48]. Furthermore, even in small lesions, it may allow precise tumor volume delineation for radiation therapy and help differentiating tumor progression from treatment effects.

FAPI ligands usually do not cross the intact BBB [49], but the initial BBB disruption is a hallmark of Central Nervous System (CNS) metastasis [50]. In brain metastases, FAP expression is elevated, primarily in stromal cells within collagen-rich areas [47]. Tracer uptake depends not only on FAP expression in the tumor stroma but also on BBB permeability, as FAPI tracers exhibit reduced penetration in areas with an intact BBB [18]. BBB disruption in brain metastases, often caused by tumor neovascularization, leads to abnormal blood vessels, damaged endothelial cells, thickenings of basement membranes, impairs of intercellular tight junctions, and increased fenestrae and pinocytotic vesicles. This phoenomena facilitate greater tracer penetration, as shown by increased permeability to fluorescent tracers in metastatic lesions [51]. Inflammation and treatment effects can worsen BBB disruption. For example, pseudo-progression and radiation necrosis involve tissue inflammation, edema, higher vascular permeability, and BBB breakdown, allowing FAPI uptake in regions with BBB leakage [52]. This disruption may restrict FAPI tracer penetration and TBR in early-stage metastases with intact BBB, where tracers struggle to cross effectively, but it can promote higher uptake and TBR in later stages [52, 53].

### Limitations

Despite the existing evidence, several limitations need to be disclosed. Many studies had constrained sample sizes, with some studies including only a few cases, which affects the generalizability of the findings and potentially affects the robustness of diagnostic accuracy estimates for PET imaging; this necessitates the need for further studies with a primary focus on brain metastatic lesions with enough sample sizes for each tumor type and subtype. Moreover, the current evidence is primarily radiological, and the lack of histopathological confirmation raises concerns about false positives, as specific lesions could be inflammatory or benign diseases; diagnoses often rely on follow-up imaging or clinical correlation. Another limitation is the publication bias, which, via Egger’s test and trim-and-fill adjustment, may materially change the pooled estimates.

### Influence of primary tumor type and future directions

The diagnostic performance of FAPI and [18F]FDG PET/CT in detecting brain metastases varies by primary tumor. FAPI PET/CT shows higher detection rates in some cancers, but in some other cancers, its performance is comparable. These findings suggest that the benefits of FAPI imaging may be more evident in tumor types characterized by low metabolic activity or in anatomical regions with high physiological [^18^F]FDG uptake, where conventional PET imaging tends to be less effective. The variability of [^18^F]FDG PET/CT based on tumor histology (grading) and metabolic behavior, which can lead to false-positive and false-negative findings, can support the need for alternative tracers, such as FAPI. Further studies are needed to better evaluate FAPI’s utility in specific cancer types and clinical settings, particularly through prospective randomized trials that can provide Level 1 evidence of its value. Ongoing research into FAPI’s prognostic value and its role in treatment response assessment will further clarify its position in the evolving landscape of precision oncology.

Future research needs to focus on larger, multicenter studies to confirm the efficacy of FAPI-based PET/CT in various tumor categories, focused on histopathological confirmation of FAP to precisely evaluate detected lesions and allow for comparison of FAPI-based PET/CT. Investigating the correlation between FAPI uptake and histopathological markers of BBB disruption and fibrotic stromal activity could be a valuable goal. Additionally, standardizing imaging protocols and interpretation criteria is essential to maintain consistency.

## Conclusion

FAPI-based PET/CT has the potential to serve as a key step in identifying brain metastatic lesions, offering improved detection rates and contrast compared to [^18^F]FDG PET/CT. Its minimal background uptake in the brain tissue enhances the visibility of lesions, making it an essential tool for early detection and treatment planning. However, these findings are preliminary and necessitate further studies.

## Data Availability

All data generated or analysed during this study are included in the manuscript. The datasets extracted from the included studies and the analysis code are available from the corresponding author upon reasonable request. All original source data are available from the cited publications.
